# The Crystalline Structure of Thin Bismuth Layers Grown on Silicon (111) Substrates

**DOI:** 10.3390/ma15144847

**Published:** 2022-07-12

**Authors:** Sandra Stanionytė, Tadas Malinauskas, Gediminas Niaura, Martynas Skapas, Jan Devenson, Arūnas Krotkus

**Affiliations:** 1 Center for Physical Sciences and Technology, Saulėtekio av. 3, LT-10257 Vilnius, Lithuania; gediminas.niaura@ftmc.lt (G.N.); martynas.skapas@ftmc.lt (M.S.); jan.devenson@ftmc.lt (J.D.); arunas.krotkus@ftmc.lt (A.K.); 2 Institute of Photonics and Nanotechnology, Vilnius University, Sauletekio av. 3, LT-10257 Vilnius, Lithuania; tadas.malinauskas@ff.vu.lt

**Keywords:** bismuth thin film, molecular beam epitaxy, high-resolution X-ray diffraction

## Abstract

Bismuth films with thicknesses between 6 and ∼30 nm were grown on Si (111) substrate by molecular beam epitaxy (MBE). Two main phases of bismuth — α-Bi and β-Bi — were identified from high-resolution X-ray diffraction (XRD) measurements. The crystal structure dependencies on the layer thicknesses of these films were analyzed. β-Bi layers were epitaxial and homogenous in lateral regions that are greater than 200 nm despite the layer thickness. Further, an increase in in-plane 2θ values showed the biaxial compressive strain. For comparison, α-Bi layers are misoriented in six in-plane directions and have β-Bi inserts in thicker layers. That leads to smaller (about 60 nm) lateral crystallites which are compressively strained in all three directions. Raman measurement confirmed the XRD results. The blue-sift of Raman signals compared with bulk Bi crystals occurs due to the phonon confinement effect, which is larger in the thinnest α-Bi layers due to higher compression.

## 1. Introduction

Bismuth (Bi) is a semimetal with unique physical properties. Its electron energy dispersion is very anisotropic, the effective masses of the carriers are small, and their free-flight distances are large. When the Bi layer is thinned to approximately 30 nm, it is converted from a semimetal to a semiconductor [1]. Bi nanowires can also become semiconducting when their diameter is below 60 nm [2]. Interest in thin Bi layers has grown in particular recently, as it has become clear that a few atomic layers of the thick structures of this material can become topological insulators [3,4]. This variety of bismuth phases has even led to it being seen as the most important electronic material of the future [5]. In addition, nanometre-thin bismuth layers are being investigated for many different applications, such as sensors [6], thermoelectricity [7], contacts for Na-ion batteries [8], femtosecond optical switches [9], and so on.

Various technologies were used to obtain high-quality Bi layers: thermal evaporation [10], electrodeposition [11], magnetron sputtering [6], pulsed laser deposition [12], and molecular beam epitaxy (MBE) [13]. Epitaxial Bi layers were grown by MBE on a variety of substrates, such as graphene [14], highly oriented pyrolytic graphite [15], NaCl [6], InAs [16], SiC [17], and silicon [13]. Compatibility with existing silicon technology, the ability to grow full wafer-sized homogeneous layers of several nanometers thickness that can be transferred to other secondary substrates, has made Si (111) substrates [18] the most popular for growing Bi layers. However, even on the (111)-oriented silicon substrates, the Bi layers do not always grow in the same way. Depending on the technological conditions, the growth starting from individual islands of the Stranski–Krastanov type or continuous layer-by-layer growth is possible. In the second case, we obtain a homogeneous, hexagonal symmetry layer whose c-axis coincides with the [111] direction of the silicon substrate. Such layers are commonly referred to as β-Bi or Bi (111). In the first case, the growth begins parallel to the three equivalent <1$\bar{1}$0> directions of the Si substrate, so there are several possibilities of the in-plane arrangement relative to the substrate and textured layers are obtained. The resulting layers are named α-Bi or Bi (110).

The layers of both of these phases differ in some physical properties important for their potential applications. THz pulse-emission studies show that the electrical characteristics of β-Bi show a pronounced anisotropy in the layer plane; in the case of α-Bi, such an anisotropy does not exist [19]. As one of the heavy elements, Bi is a common constituent of many well-known topological insulators because of its strong spin-orbital coupling strength. In particular, the β-Bi bilayer film was theoretically predicted as a promising candidate for a two-dimensional topological insulator [20]. On the other hand, when several bilayers thick, α-Bi can become a nontrivial two-dimensional topological insulator due to the substrate-induced strain and charge doping [21].

In the present contribution, we investigated how the crystalline structure of both bismuth phases changes with the varying thickness of Bi layers. Extensive measurements using X-ray diffraction analysis (XRD), transmission electron microscopy (TEM), as well as the Raman spectra analysis, have shown that both α-Bi and β-Bi thin films grown on Si (111) substrates are stressed, but in the first case, these stresses relax with increasing layer thickness faster than in the second case.

## 2. Growth and Experimental Details

Bi samples were grown using a solid source Veeco GEN Xplor MBE system, equipped with a conventional Dual Filament bismuth source on (111)-oriented Si substrates. The thermal desorption of native oxide from the Si (111) surface has been performed at ∼1100 °C temperature according to thermocouple readings prior to the growth. The 7 × 7 surface reconstruction corresponding pattern appearance on the RHEED screen indicated completion of the oxide removal process, and the substrate temperature controller settings were decreased to the projected Bi growth temperature. After the thermocouple temperature readings had reached the temperature set in the temperature controller, a 30 min pause was used to stabilize the substrate temperature. The main shutter was kept closed at that time, and the Bi source shutter was open to stabilize the molecular Bi flux and to measure its beam equivalent pressure (BEP). After that, keeping the Bi shutter open, the main shutter was opened for the entire growth period. After closing the Bi shutter, the substrate was immediately removed from the growth chamber to minimize the mobility of the surface atoms.

The substrate temperature was controlled by a thermocouple. A kSA BandiT substrate temperature measurement tool based on the temperature-dependent optical absorption edge measurement, which was installed into the MBE reactor, was not able to measure temperatures below ∼200 °C as the main light source in the transmittance spectrum measurement mode is a substrate heater that at lower temperatures flashes with an uncertain period to provide the heat to the wafer holder and the substrate. The BandiT optical absorption edge-based temperature readings at the same thermocouple temperature readings varied within the 30 °C range. Assuming that the optical absorption edge measurement is a more accurate temperature determination method than the contactless thermocouple-based measurement, it is reasonable to assume that the substrate temperature at lower than 200 °C temperatures will also strongly depend on the unique substrate holder optical properties. Hence, the temperature measurement accuracy in this experiment is likely to be within the 30 °C range or even worse. This may cause some uncertainty when evaluating the growth temperature influence on the crystalline structure of the grown sample.

The main growing conditions of the different Bi samples are shown in Table 1. It was assumed that the growth temperature is the main factor determining the phase of the grown bismuth. To clarify the influence of the growth temperature on the phase formation, two samples were grown at very different temperatures. Sample S01 has been grown at a relatively high 100 °C according to the thermocouple readings while sample S06 has been grown at a temperature below 0 °C. The temperature controller installed on the MBE reactor is not able to measure negative °C scale temperatures. The growth temperature of sample S06 can be roughly estimated from the observed temperature drop rate. In our estimation from the exponential temperature decay, it can be between −30 and −15 °C. The structural analysis of these two samples confirmed the formation of different Bi phases. The higher growth temperature induces formation of a β-Bi phase while the lower growth temperature promotes the α-Bi phase formation.

The structure of the layers was characterized using X-ray diffraction (XRD) out-of-plane ω/2θ, in-plane 2θχ/ϕ, and ϕ measurements. The thicknesses were determined by the X-ray reflectivity (XRR) method. These measurements were performed using the Smartlab (Rigaku, Japan) diffractometer with a 9 kW rotating Cu anode X-ray generator equipped with an in-plane arm. Ge(400)x2 monochromator was used for K_α1_ line separation, and scintillation SC-70 (0D) detector for signal registration. For angular resolution improvement in in-plane measurements, Soller slits and parallel slit analyzers were used. The main structural parameters of Bi samples from XRD measurements are shown in Table 1.

TEM measurements were carried out by FEI Tecnai G2 F20 X-TWIN (Thermo Scientific, Eindhoven, The Netherlands). TEM operates at 200 kV with a STEM module equipped with an EDX detector for elemental mapping and a high-angle annular dark-field (HAADF) detector for the Z-contrast imaging. For bright-field imaging, a 10 μm diameter objective aperture was used for enhanced contrast. Cross-sectional TEM specimens were prepared by focused Ga-ion beam using FEI Helios Nanolab 650 dual beam microscope equipped with an Omniprobe manipulator.

Raman spectra were recorded by an inVia Raman (Renishaw, Wotton-under Edge, UK) spectrometer equipped with a thermoelectrically cooled to −70 °C CCD camera and microscope. The 633 nm laser beam of the He-Ne laser was used for the excitation of the spectra. The scattering geometry was 180°. To avoid laser radiation-induced degradation of the sample, the laser power was restricted to 0.05 mW. The 50×/0.75 NA objective lens and 2400 lines/mm grating were employed to record the Raman spectra. The spectral slit width determined as the FWHM of the emission line from the Neon lamp was found to be 2.5 cm^−1^ at 183 cm^−1^. The Raman scattering wavenumber axis was calibrated by recording the spectrum of sulfur. The integration time was 10 s, and each spectrum was recorded by an accumulation of 40 scans. Additionally, four spectra were averaged, yielding a total integration time of 1600 s. The parameters (peak position, intensity, and full width at half maximum, FWHM) of the vibrational bands were determined by fitting the experimental spectra with Gaussian–Lorentzian form components using GRAMS/A1 8.0 software (Thermo Scientific, Waltham, MA, USA).

## 3. Results and Discussion

Bulk bismuth crystalizes in a rhombohedral structure (space group R-3m, No. 166). Its unit cell has two atoms and can be described with two parameters: the lattice constant *a* and angle between axes α. It is also possible to describe the lattice equivalently in a hexagonal notation with the parameters a_hex_ and c_hex_. Hexagonal notation is more convenient for the evaluation of strain while growth direction coincides with [111]rhombohedral or [0003] hexagonal directions because tension-compression movement depends on c_hex_/a_hex_ variation. As to avoid mix-ups in lattice plane notations, hereafter, mainly the rhombohedral indexing will be used.

β-Bi atoms are arranged in bilayers, and each atom is covalently bonded with the three nearest atoms. By much weaker bonds, they are also bonded with three other second-nearest atoms, so the β-Bi film behaves like a van der Waals material. On the surface, a honeycomb lattice is formed, similar to graphene. Though the honeycomb is buckled because the six atoms belong to different layers of the Bi bilayer. Thus only three mirror planes are present. In the case of α-Bi, atoms are also arranged in bilayers, but the vertical separation of the two atoms in the bilayer is much smaller (it seems like puckered single layer), and each atom is covalently bonded with the two nearest atoms. The third bond is a dangling bond that pairs puckered layers to form a black-phosphorus-like structure with a pseudo-square surface with a single mirror plane [22,23,24,25].

### 3.1. X-ray Measurements

**Figure 1:**
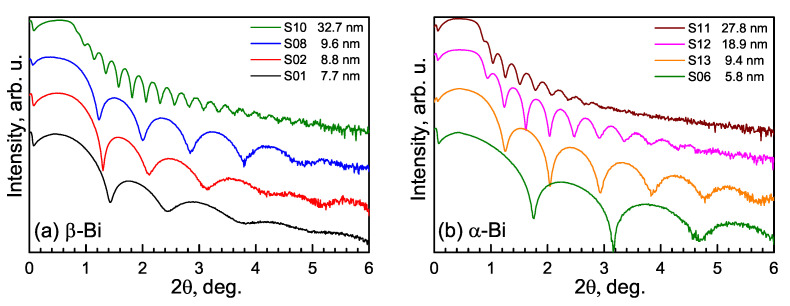
The X-ray reflectivity curves of β-Bi (**a**) and α-Bi (**b**) samples.

Samples with four thicknesses of each bismuth phase were investigated. Their thicknesses were determined by the XRR method, and the results are shown in Figure 1. The thicknesses of all the layers vary from ∼6 up to ∼30 nm. The smaller distance between interference peaks corresponds to higher thickness. The presence of the fringes up to 6 degrees shows low roughness of the layer surface for all structures.

The structure and crystalline orientations of Bi samples were analyzed by using XRD out-of-plane ω/2θ and in-plane 2θχ/ϕ measurements. The two main surface orientations of rhombohedral bismuth, β-Bi and α-Bi and their in-plane relation to the substrate crystallographic axes were identified.

**Figure 2:**
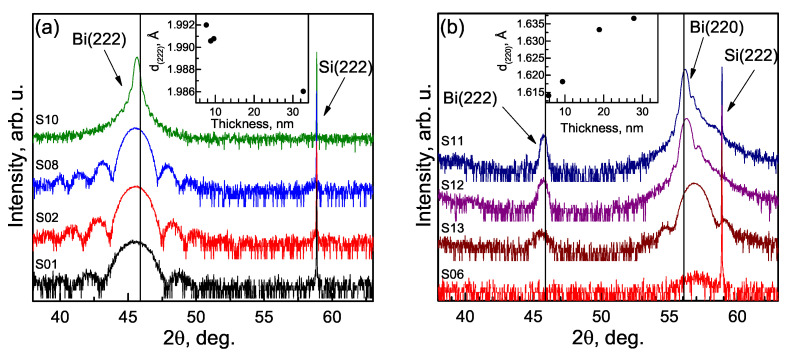
Out-of-plane ω/2θ curves of β-Bi (**a**) and α-Bi (**b**). The vertical black lines show 2θ values of Bi (222) and Bi (220) planes calculated for lattice with *a* = 4.7459 Å and α = 57.237. Interplanar spacing dependencies on the layer thickness are shown in the insets.

In the first set of samples, ω/2θ curves of β-Bi samples showed peaks around 22.3°, 45.5°, and 71.0° corresponding to a pure β-Bi orientation; the existence of other orientations was not noticed in this set of samples. In Figure 2a, the (222) peaks of β-Bi layers with different thicknesses are shown. 2θ values in out-of-plane measurements are slightly lower than calculated for lattice with *a* = 4.7459 Å and α = 57.237 showing larger than theoretical interplanar spacing in the growth direction (Figure 2a, inset).

The second set of samples has a preferable α-Bi orientation with peaks around 27.5° and 56.2°. The 2θ values of α-Bi, in this case, are slightly higher than theoretical, decreasing toward theoretical with increases in the thickness of the layer (Figure 2b, right peak). Increasing interplanar spacing (Figure 2b, inset) in a growth direction and the asymmetric shape of the peaks confirm the lattice relaxation of the thicker films. In the thinnest sample, S06, solely α-Bi orientation is identified, but the XRD traces of thicker samples from this set contain additional peaks corresponding to β-Bi orientation. These Bi (222) peaks shift to the right, as in the case of pure β-Bi type layers; their amplitude becomes higher in thicker layers evidencing a higher amount of additional orientation material (Figure 2b, left peak).

**Figure 3:**
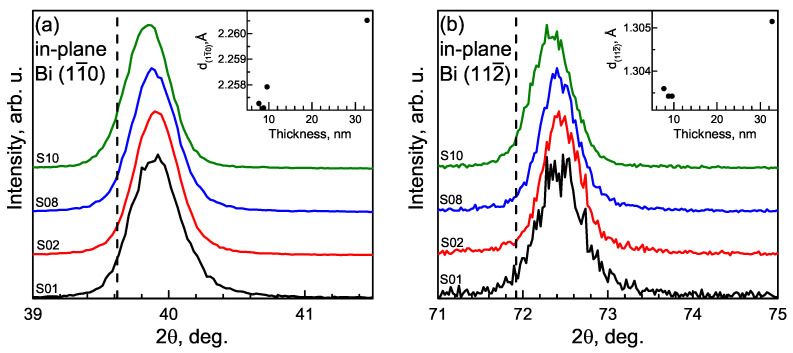
In-plane 2θχ/ϕ measurements of (1$\bar{1}$0) (**a**) and (11$\bar{2}$) (**b**) β-Bi planes. The vertical dashed lines show theoretical 2θ values for the planes mentioned above. Interplanar spacing dependencies on the layer thickness are shown in the insets.

In-plane measurement of β-Bi samples shows the opposite strain to out-of-plane measurements: peaks corresponding to (1$\bar{1}$0) and (11$\bar{2}$) planes perpendicular to the surface have higher 2θ values (lower interplanar distance—insets of Figure 3a,b), showing that biaxial compressive strain is perpendicular to growth direction (Figure 3a,b).

The values of strain that are listed in Table 2 were calculated according to the formula: (dmeas− d_theor_)/d_theor_, where d_meas_ and d_theor_ are the measured and the theoretical interplanar distances of the plane. Both out-of-plane and in-plane strain decreases with increasing thicknesses of the layer, showing that the structure relaxes from compressive biaxial strain. The origin of the compressive strain is a huge lattice mismatch: the interplanar distance between Bi planes is ∼18% larger than Si, but the lower than expected strain value could be explained by the formation of the van der Waals bonds between the film and substrate (and/or between atomic bilayers of bismuth).

**Figure 4:**
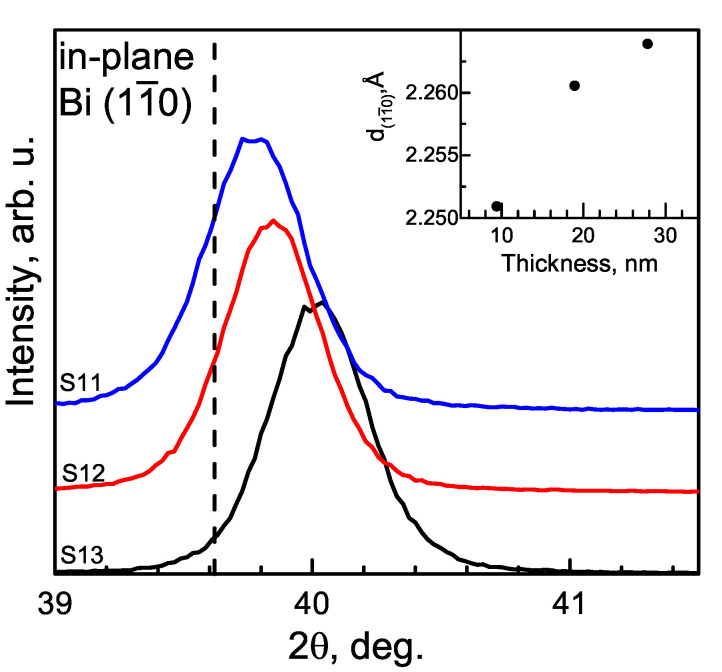
In-plane 2θχ/ϕ measurement of the α-Bi (1$\bar{1}$0) plane. The vertical dashed lines show theoretical 2θ values for the planes mentioned above. Interplanar spacing dependencies on the layer thickness are shown in the insets.

In the α-Bi case, in-plane measurements showed the same effect as in the out-of-plane measurements, so out-of-plane and in-plane interplanar spacings increase with an increasing layer thickness (Figure 4). However, for the thickest sample, S11, the unit cell volume is close to its theoretical value, but a and α parameters are higher and lower, respectively. The explanation for such behavior could be lattice distortion to reduce strain. The in-plane and out-of-plane strain values are given in Table 3.

**Figure 5:**
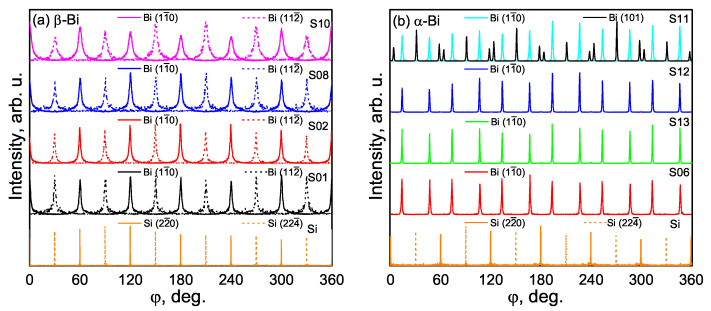
In-plane ϕ scans of (1$\bar{1}$0) (solid lines) and (11$\bar{2}$) (dashed lines) planes for β-Bi (**a**) and in-plane ϕ scans of (1$\bar{1}$0) and (101) (to avoid confusion is shown only for S11) planes for α-Bi (**b**). Si (2$\bar{2}$0) (solid lines) and Si (22$\bar{4}$) (dashed lines) ϕ scans are given in both pictures for the evaluation of the azimuthal orientation between the substrate and the layer.

To understand the relationship between the substrate and the preferential growth of the β-Bi film for this set of samples, ϕ scans of Bi (1$\bar{1}$0), Bi (11$\bar{2}$), Si (2$\bar{2}$0), and Si (22$\bar{4}$) were measured. All the scans for β-Bi and Si give six peaks because there are three planes perpendicular to the surface measured from both sides (Figure 5a). Solid lines correspond to the repeatability of Bi (1$\bar{1}$0) and Si (2$\bar{2}$0) planes, and the dashed lines correspond to Bi (11$\bar{2}$) and Si (22$\bar{4}$) planes. The coincidence of Bi (1$\bar{1}$0) and Si (2$\bar{2}$0) ϕ angles means that these planes are parallel, and both make an angle of 30° with the second pair of parallel Bi (11$\bar{2}$) and Si (22$\bar{4}$) planes. Growth in such a relationship is preferable due to the lowest possible lattice mismatch and similar hexagonal arrangement of the atoms. Moreover, this result proves that Bi (111) layers have a single in-plane orientation.

For evaluation of the azimuthal orientation between the substrate and α-Bi, ϕ scans of Bi (1$\bar{1}$0) planes perpendicular to the surface were measured. In Figure 5b, 12 peaks from measured planes can be seen. Since the Bi (110) orientation has only one (1$\bar{1}$0) plane perpendicular to the sample’s surface, the 12 observed reflections from this plane prove that 6 in-plane orientations are present in these samples. Further, Bi (1$\bar{1}$0) planes are parallel neither to Si (2$\bar{2}$0) nor to (22$\bar{4}$) planes like in β-Bi.

**Figure 6:**
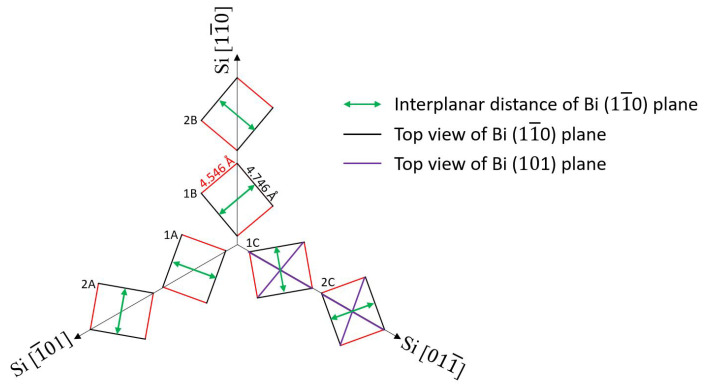
Scheme of six in-plane orientations of α-Bi in respect to Si (111) substrate <1$\bar{1}$0> directions. Rectangles represent the (110) plane of bismuth unit cell laying on the Si (111) plane.

In Figure 6, six in-plane orientations of α-Bi in respect to the Si (111) substrate <1$\bar{1}$0> directions are shown. Rectangles represent the (110) plane of the bismuth unit cell laying on the Si (111) plane. These rectangular unit cells with edges equal to 4.546 Å (red) and 4.746 Å (black) for shorter and longer edges, respectively, are arranged in all possible ways that one of their diagonals (purple) coincides with Si <1$\bar{1}$0> directions. Perpendicular to the surface, the Bi (101) plane is parallel to these diagonals. Knowing that the Si (22$\bar{4}$) plane is also perpendicular to the surface and parallel to the Si [1$\bar{1}$0] direction, we can conclude that the Bi (101) plane is parallel to the Si (22$\bar{4}$) plane. This is confirmed by in-plane XRD measurements (Figure 5b, black solid line), where six of the most intensive peaks coincide with the Si (22$\bar{4}$) peaks (the smaller peaks belong to secondary, non-coinciding diagonals). The crystallites with the same azimuthal orientation of α-Bi on Si (111) were observed in the STM investigation in [26].

From Figure 6, the origin of the observed pseudo-12-fold symmetry from Bi (1$\bar{1}$0) planes becomes clearer. These perpendicular to surface planes are parallel to a longer rectangular unit cell edge (Figure 6, black edge of rectangular), the interplanar distance between them is marked with a green arrow. These planes are responsible for X-ray reflections at the angles of 13.8°, 46.2°, 73.8°, 106.2°, 133.8°, and 166.2° (and at six by 180° larger angles for reflections from the other sides of the planes). This proves that there are two sets (Figure 61A–C and 2A–C) of three-fold rotational axes that differ by 27.6°, which arrange so that the Si <1$\bar{1}$0> direction makes 16.2° angles with the Bi (1$\bar{1}$0) plane.

### 3.2. TEM Measurements

**Figure 7:**
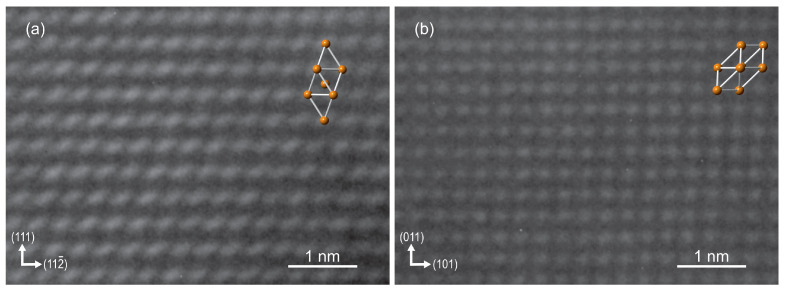
High-resolution TEM micrographs of samples S10 (**a**) and S11 (**b**) with superimposed Bi lattice models. Determined zone axes of the Bi layer are [$\bar{1}$10] and [11$\bar{1}$] for S10 and S11, respectively. The Si substrate zone axis was determined as [$\bar{1}$10].

The two thickest layers, S10 (β-Bi) and S11 (α-Bi), were thoroughly investigated by TEM. These samples were prepared with a focused Ga-ion beam perpendicular to one of the Si <1$\bar{1}$0> directions. High-resolution TEM micrographs of both β-Bi and α-Bi samples are presented in Figure 7a and Figure 7b, respectively. During measurement, the samples were oriented in such a way that the zone axis of Si was set to [$\bar{1}$10]. The zone axes of Bi in samples S10 and S11 were determined as [$\bar{1}$10] and [11$\bar{1}$], respectively. Arrows indicate the direction of the corresponding plane normal; that is, Bi (11$\bar{2}$) in S10 and Bi (101) in S11 are parallel to Si (22$\bar{4}$). This complements the XRD in-plane measurement results that these Bi planes are parallel to the corresponding Si planes. Superimposed on both micrographs are elementary cells of corresponding lattice models for β-Bi and α-Bi for clarity. The orderly arrangement of atoms in the Bi layers has been observed from the beginning of growth; in both cases, there was no initial wetting layer, as discussed in [21]. In both cases, the thickness of the layers was about 30 nm, which was in good agreement with the thicknesses obtained from the XRR measurements.

**Figure 8:**
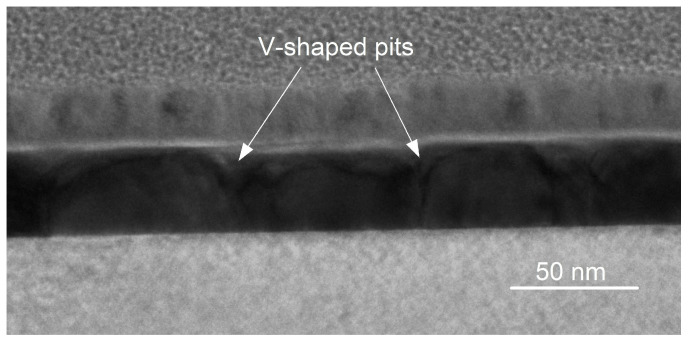
Bright-field TEM micrograph of sample S11. Arrows indicate locations of V-shaped pits that are formed between individual crystallites.

The lateral crystallite size of sample S10 is more than 200 nm, while in sample S11, it is determined to be ∼60 nm. Figure 8 shows the presence of V-shaped pits between these crystallites on the Bi layer surface. Their density is ∼2.6 × 10^6^ cm^−1^.

### 3.3. Raman Analysis

Raman spectroscopy can provide information on the local structure and crystallinity of Bi nanoparticles and thin films [10,27,28,29,30,31]. In this work, we have analyzed film thickness-induced spectral changes for β-Bi and α-Bi samples.

**Figure 9:**
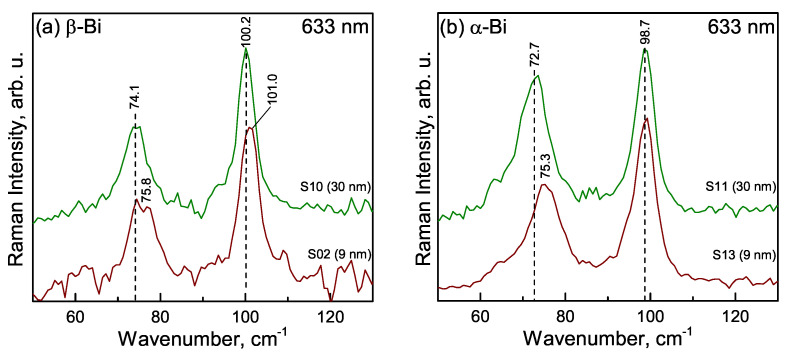
Raman spectra of 9- and 30-nm thick β-Bi (**a**) and α-Bi (**b**). Intensities are normalized to the intensity of the A_1g_ mode and spectra are shifted vertically for clarity.

Figure 9 compares the Raman spectra of thin Bi films of different thicknesses. The two first-order Raman modes of the Bi bulk E_g_ and A_1g_ are at 69 cm^−1^ and 94 cm^−1^, respectively [10]; in bismuth nanostructures, these peaks experience a significant blue-shift [27,28]. The structure of the thin bismuth layer might be affected by laser radiation-induced disordering and oxidation [29,30]. To eliminate possible laser radiation effects, extremely low (0.05 mW) laser power was employed. No Raman bands related to bismuth oxidation products were observed. The parameters of the Raman bands are listed in Table 4.

Comparison of spectral parameters of 30 nm-thick bismuth films prepared in different growing conditions (α-Bi and β-Bi samples) reveals three clear differences: (i) intensity ratio A_Eg_/A_A1g_ increases in the case of α-Bi, (ii) peak wavenumbers are blue-shifted in the spectrum of sample β-Bi, and (iii) considerable narrowing of both spectral bands is visible in the spectrum of sample β-Bi — lower full width at a half maximum (FWHM) values (Table 4). Narrowing of the bands suggests higher structural ordering in the case of sample β-Bi. The analysis of vibrational frequencies of the bands suggests that the structure of the studied Bi-films deviates from rhombohedral because wavenumbers are higher compared with values observed for rhombohedral Bi powder (69.0 and 94.5 cm^−1^, for E_g_ and A_1g_ modes, respectively) [10].

A comparison of Bi films with different thicknesses revealed an upshift in the frequency of the E_g_ mode and the broadening of both bands for thinner samples. The observed blue shift is consistent with the phonon confinement model [31]. The model predicts a higher frequency upshift for E_g_ phonon compared with A_1g_, in agreement with this work’s observations. However, a comparison of 9-nm thin films of different structures (α-Bi and β-Bi) disclosed the higher near-field crystalline ordering for the later sample because of lower FWHM values (Table 4). The frequency upshift for the E_g_ mode comparing 30- and 9-nm films was found to be 2.6 and 1.7 cm^−1^ for α-Bi and β-Bi samples, respectively. The higher frequency upshift in the case of α-Bi might be related to increased compressive stress for a thinner film. In addition, the presented spectral data indicate an increase in relative intensity of A_1g_ phonon for the β-Bi sample and with a decrease in the film thickness (Table 4). Thus, the highest relative intensity of the A_1g_ band was detected for the 9-nm thick β-Bi film.

## 4. Conclusions

In summary, bismuth layers with thicknesses ranging from 6 nm to approximately 30 nm were grown the molecular beam epitaxy on (111)-oriented Si substrates. Depending on the growth conditions, two types of Bi layers were obtained: homogeneous hexagonal symmetry β-Bi layers and α-Bi layers consisting of rhombohedral crystallites oriented along with three equivalent Si <$\bar{1}$10> directions. In α-Bi, the gaps between the crystallites are filled with β-Bi inserts. All samples were examined in detail using X-ray diffraction, transmission electron microscopy, and Raman spectroscopy measurements.

In-plane and out-of-plane X-ray measurements have evidenced that α-Bi is compressively strained in all three directions, and this strain decreases with increasing layers‘ thicknesses due to their partial relaxation. In contrast, β-Bi layers are biaxially compressed in the layer plane, but their interatomic distances increase in the direction perpendicular to the layer plane. Both of these effects decrease with increasing layer thickness. The TEM measurements show that all layers have an orderly crystalline structure from the beginning of growth; no wetting layers were observed. The characteristic lateral dimensions of the α-Bi crystallites at the substrate are about 60 nm; homogeneous β-Bi regions are greater than 200 nm.

Raman signals of the bismuth layers are all blue-shifted from their positions in the bulk bismuth crystals due to the phonon confinement. This effect is enhanced when thinning the layers, but in α-Bi, this enhancement is larger than in β-Bi, possibly due to a greater overall compression in the former material. The linewidths of the Raman spectra in α-Bi are larger than in β-Bi, which is understandable knowing the results of the X-ray measurements. In the first case, we have a textured structure with β-Bi inclusions, while in the second case, the layer is homogeneous. 

## Figures and Tables

**Table 1:** The growth conditions and main structural parameters of the samples.

Sample	Thickness, nm	Phase	Growth Temp., °C	Bi BEP, Torr	Growth Duration, s
S01	7.7	β-Bi	100	7.58–8.09 × 10^−8^	706
S02	8.8	β-Bi	21	4.46–4.607 × 10^−8^	706
S08	9.6	β-Bi	50	5.764 × 10^−8^	1080
S10	32.7	β-Bi	50 ÷ 55	5.75 × 10^−8^	3940
S06	5.8	α-Bi	−30 ÷ −15	1.8 × 10^−8^	2440
S13	9.4	α-Bi	50	7.8 × 10^−8^	900
S12	18.9	α-Bi	50	7.8 × 10^−8^	1870
S11	27.8	α-Bi	50	7.8 × 10^−8^	2700

**Table 2:** Strain calculated from three planes for β-Bi layers with different thicknesses.

Sample	Strain Calculated from d_(222)_ (ϵ_zz_), %	Strain Calculated from d_(1$\bar{1}$0)_ (ϵ_xx_), %	Strain Calculated from d_(11$\bar{2}$)_ (ϵ_yy_), %
S01	0.82	−0.69	−0.63
S02	0.75	−0.69	−0.65
S08	0.76	−0.66	−0.65
S10	0.52	−0.54	−0.51

**Table 3:** Strain calculated from two planes for α-Bi layers with different thicknesses.

Sample	Strain Calculated from d_(220)_ (ϵ_zz_), %	Strain Calculated from d_(1$\bar{1}$0)_ (ϵ_xx_), %
S13	−1.26	−0.96
S12	−0.34	−0.54
S11	−0.13	−0.39

**Table 4:** Parameters of Raman bands of Bi thin films grown at different conditions.

Sample	Raman Mode	ν, cm^−1^	FWHM	A_E1g_/A_A1g_
S11	E_g_	72.7	8.1	1.36
	A_1g_	98.7	5.3	
S13	E_g_	75.3	8.9	1.00
	A_1g_	98.7	6.0	
S10	E_g_	74.1	6.9	0.83
	A_1g_	100.2	4.6	
S02	E_g_	75.8	7.4	0.58
	A_1g_	101.0	5.2	
Bulk Bi [10]	E_g_	69		
	A_1g_	94		

## Data Availability

Not applicable.
